# Perioperative Respiratory Adverse Events Among Pediatric Surgical Patients in University Hospitals in Northwest Ethiopia; A Prospective Observational Study

**DOI:** 10.3389/fped.2022.827663

**Published:** 2022-02-11

**Authors:** Desalegn Muche Wudineh, Yophtahe Woldegerima Berhe, Wubie Birlie Chekol, Habtu Adane, Misganaw Mengie Workie

**Affiliations:** ^1^Felege-Hiwot Referral Hospital, Bahirdar, Ethiopia; ^2^Department of Anesthesia, University of Gondar, Gondar, Ethiopia

**Keywords:** perioperative respiratory adverse events, perioperative complications, pediatric anesthesia, general anesthesia, adverse events

## Abstract

**Introduction:**

Perioperative respiratory adverse events (PRAEs) are frequent among pediatrics surgical patients and are accountable for 3/4^th^ of perioperative critical incidents and 1/3^rd^ of cardiac arrests.

**Objective:**

Assess the prevalence and factors associated with PRAEs among pediatric surgical patients in University Hospitals in Northwest Ethiopia, 2020.

**Methodology:**

After ethical approval obtained prospective observational study was conducted among 210 pediatric surgical patients. Perioperative respiratory adverse events were defined as the occurrence of any episode of single/combination of coughing, breath holding, hypoxemia, laryngospasm and bronchospasm. Bivariate and multivariate binary logistic regression analyses were performed and variables with *p* < 0.05 at 95% confidence interval were considered as statistically significant.

**Results:**

The prevalence of PRAEs was 26.2% (CI: 20.5–30.9%). A total of 129 episodes of PRAEs were occurred and of them, 89 (69.0%) were occurred in the postoperative period. Desaturation was the predominant adverse event which was observed 61 (47.3%) times. Age <1 year (AOR: 3.6, CI: 1.3–10.0), ASA ≥ 3 (AOR: 5.2, CI: 1.9–22.9), upper respiratory tract infections (URTIs) (AOR: 7.6, CI: 1.9–30.2), secretions in the upper airway (AOR: 4.8, CI: 1.4–15.9) and airway related surgery (AOR: 6.0, CI: 1.5–24.1) were significantly associated with PRAEs.

**Conclusions:**

Prevalence of PRAEs was high among pediatric surgical patients; the postoperative period was the most critical time for the occurrence of PRAEs and desaturation was the commonest PRAE. Age <1 year, URTIs (recent or active), secretions in the upper airways, ASA ≥ 3 and airway related surgery were significantly associated with PRAEs. Clinicians should perform effective risk assessment, preoperative optimization and preparation for the management of PRAEs.

## Introduction

Adverse event is defined as unanticipated and unwanted response to medical intervention during the perioperative period that can threaten patient wellbeing. Respiratory adverse events are any episodes of desaturation, partial or complete airway obstruction, persistent coughing, breath holding and bronchospasm (1–3). Despite improvements in pediatrics anesthesia, respiratory adverse events are still the most frequent cause of serious morbidity and mortality in the perioperative period. The 3/4^th^ of all critical incidents and the 1/3^rd^ of all perioperative cardiac arrests in pediatric anesthesia are caused by respiratory adverse events. Preoperative identification of those children at high risk is a challenging process (4, 5). Children are vulnerable to respiratory adverse events because of anatomical and physiological considerations and frequent respiratory tract infections (URTIs). The most common respiratory adverse events under anesthesia are desaturation, breath holding, laryngospasm, bronchospasm and coughing. Among these, laryngospasm, bronchospasm and persistent hypoxemia could result potentially devastating complication and death (6–8).

Inconsistent definitions of respiratory adverse events among studies make comparisons difficult and results in discrepancy on magnitude of the actual events (1). Despite introduction of new monitoring modalities, pharmacological products and new clinical practice guidelines, the prevalence of respiratory adverse events has remained high and increased length of hospital stay and medical costs (1, 9). The patient's age, techniques of anesthesia induction, comorbidities and airway surgery were strongly associated with increased occurrence of adverse events during surgery. Occurrence of respiratory adverse events differs according to timing of extubation whether awake or deep plane of anesthesia, airway device and urgency of procedures (10, 11). The severity of PRAEs and their complications depends on the nature of surgery, presence comorbidity, early detection, and prompt corrective measures. It may range from transient damage with full recovery to unanticipated morbidity and mortality (2). Risk assessment in the preoperative period is very important to decrease respiratory adverse events but still patient identification and selection are real challenges. If effective measures are not taken timely, serious complications and death would prevail. “*Critical respiratory incident recording and reporting system is poor particularly in the developing countries due to multiple reasons”* (12). The general objective of the current study was to assess the prevalence and factors associated with PRAEs among pediatrics surgical patients.

## Methodology

A prospective observational study was conducted from March 1 to May 30, 2020 at the University of Gondar Comprehensive Specialized Hospital (UoGCSH) and Tibebe-Ghion Specialized Hospital (TGSH). The hospitals are located at Gondar and Bahirdar towns respectively in the Northwest Ethiopia. The source population was all pediatric surgical patients (0–12 years of age) that underwent surgery under general anesthesia and the study population was all pediatric surgical patients that underwent surgery under general anesthesia at UoGCSH and TGSH during the study period. All pediatric surgical patients whose parents/legal care-givers were volunteer were included and patients who had severe head injury, intubated patient, acute respiratory distress, hypoxia requiring mechanical ventilation in the preoperative period, operated for more than once, and transferred to intensive care unit for mechanical ventilation after operation were excluded.

Sample size was calculated by using single population proportion formula with 50% proportion, maximum acceptable difference (d) = 5%, and 95% confidence interval and found 385. We used a reduction formula to determine achievable sample size as the surgical registries showed that only 780 and 660 pediatrics surgical procedures were performed annually at UGCSH and TGCSH respectively. In-addition, due to COVID-19 pandemic, the flow of pediatric surgical patients was reduced. After reduction, the sample size became 195 and 15% non-response rate was added and the final sample size was 225.

All eligible pediatric surgical patients who received general anesthesia were included in the study. The dependent variables were PRAEs which were measured in terms of any episode of either coughing, breath holding, hypoxemia, laryngospasm, or bronchospasm. The independent variables were patient factors (history of prematurity, age, ASA classification, family history of asthma, URTIs, and other comorbidities), anesthetic factors (techniques of anesthetic induction, type of airway device, number of intubation attempts, muscle relaxant, depth of anesthesia, perioperative opioid use, and experience of the anesthesia providers), and surgical aspects (surgical site, duration, and urgency).

### Operational Definitions

**Perioperative respiratory adverse event**: any episode of single/combination of coughing, breathe holding, hypoxemia, laryngospasm and bronchospasm (2, 3, 13–19). Detailed operational definitions were prepared accordingly.

**Laryngospasm**: complete airway obstruction with abdominal and chest muscle rigidity that require positive pressure ventilation or administration of succinylcholine (1).

**Bronchospasm**: increased work of breathing, particularly during expiration and wheezing on auscultation or requires bronchodilators (7, 13).

**Desaturation or hypoxemia**: peripheral arterial oxyhemoglobin saturation (SpO_2_) <95% more than 30 seconds measured by pulse oximetry regardless administrations of 100% Oxygen or SpO_2_ <90% in atmospheric air. Oxygen saturation was documented when pulse oximetry showed consistent readings with no artifacts (2, 7).

**Coughing**: a series of pronounced, persistent coughs lasting more than 5 s (7).

**Breath holding or apnea**: if the patient had apnea more than 15 s or irregular breathing or if the apnea is associated with bradycardia or cyanosis (13).

**Partial upper airway obstruction**: airway obstruction in the combination with snoring, inspiratory stridor or increased breathing efforts or paradoxical abdominal movement or both that can be effectively relieved by simple airway maneuvers (2).

**Multiple intubation attempts**: if required multiple intubation attempts ≥ 3 times (20). Active or current URTIs: the present of at least of two URTIs symptoms (rhinorrhea, sneezing, nasal congestion, sore or scratchy throat, cough, malaise, or fever > 38°C) at the time of surgery together with a confirmation by a parent (13).

**Active/Recent URTIs**: symptomatic URTIs during/within 2 weeks of the perioperative period was considered as active URTIs. If the URTIs occurred within 2–4 weeks before surgery but resolved at the time of surgery, it was considered as recent URTIs (13).

**Acute respiratory distress**: the presence of tachypnea, nasal flaring, grunting, intercostal and subcostal retractions, and cyanosis in room air and requires high flow Oxygen (21).

**Light anesthesia**: the presence of patient's movement with tachypnea, tachycardia and hypertension (change by more than 30% from baseline) (22).

**Oropharyngeal secretions**: the presence of secretion that requires suctioning of more than once (23).

**Pulmonary aspiration**: confirmed or suspected entrance of foreign materials such as gastric contents into the respiratory tract result in new/worsening respiratory signs such as hypoxia (24).

**Airway related surgery**: surgery that involve the airway which includes ENT and maxillofacial procedures such as adenotonsilectomy, cleft palate repair, direct laryngoscopy, and bronchoscopy (23, 25, 26).

All complications were scored according to severity from 1 (no complication) to 4 (serious complication). For patients who received anesthesia with endotracheal tubes or laryngeal mask airways, scores were multiplied by a constant factor of five. When facemask was used, scores were multiplied by a constant factor of three. Patients received a composite score of 5–20 for ETT or LMA and 3–12 facemask for each adverse event (Table 1) (23). The PRAEs were recorded starting from induction to the first postoperative hour. A patient experiencing PRAEs was counted just once independent of the numbers of events in perioperative period.

**Table 1:** Perioperative respiratory adverse events severity scoring in pediatrics patients underwent surgery under general anesthesia.

**Parameters**	**Severity score**			
	**1 (No adverse event)**	**2**	**3**	**4 (Serious adverse events)**
SpO_2_	95–100	90–94	80–89	<80
Coughing (*n*)	None	1–2	3–4	Continuous
Breath holding (seconds)	None	<15	15–30	> 30
Laryngospasm	None	Partial obstruction requires repositioning only	Partial obstruction requires CPAP	Complete obstruction requires muscle relaxant
Bronchospasm	None	Expiration phase only	Expiration and inspiration phase	Difficult to ventilate: treatment needed
Secretions	None	Minimal: no suctioning	Moderate: one suctioning	Copious: more than one suctioning

Ethical approval (**Reference No: PGC/587/07/2012**) was obtained from the Ethical Review Committee of School of Medicine, University of Gondar. Written informed consent was obtained from parents/legal care-givers of each child. When clinically significant PRAEs were noticed, data collectors reported for clinicians (Anesthetist, Surgeon or Nurse) to provide appropriate management. A pre-test was conducted on 20 (8%) patients whose data were not included in the main study. The data were analyzed by using SPSS version 20 (IBM Corporate). The normality was checked by using Shapiro-Wilk normality test. The Chi-squared or Fisher's exact tests were used when appropriate. The Hosmer-Lemeshow test was used to assess model fitness. The variance inflation factors and tolerance were used to diagnose multicollinearity. The associations between variables were determined by using bivariate and multivariate binary logistic regression. The cut-point of statistical significance was *p* < 0.2 for bivariate and <0.05 for multivariate regression at 95% confidence interval. Odds Ratio were used to describe the strength of associations.

## Results

Two hundred ten (210) pediatric surgical patients who received general anesthesia were included in this study. The response rate was 93.3% and data from 15 patients were excluded due to incompleteness. The majority of the patients 115 (54.8%) were males. The median age (inter-quartile range) was 4.0 (1.1–8.0) years. Most of the patients 189 (90%) were classified under ASA 1 and 2 while the rest were ASA 3 and above. The 23 (11%) patients had URTIs. Eighty-nine (42.4%) patients had low hemoglobin levels (<11 g/dl) preoperative. The mean duration of surgery was 82 ± 41.5 min (Table 2).

**Table 2:** Clinical characteristics of pediatric surgical patients who underwent surgery under general anesthesia in UoGCSH and TGSH from March 1–May 30, 2020 (*N* = 210).

**Variables**	**Frequency *n* (%)**	**Perioperative respiratory adverse events**	
		**Yes *n* (%)**	**No *n* (%)**
Age			
<1 year ≥ 1 year Median (IQR) (years)	52 (24.8) 158 (75.2) 4.0 (1.1–8.0)	22 (42.3) 33 (20.9)	30 (57.7) 125 (79.1)
Prematurity			
Yes No	14 (6.7) 196 (93.3)	4 (28.6) 51 (26)	10 (71.4) 145 (74)
Preoperative hemoglobin			
<11 g/dl ≥ 11 g/dl	89 (42.4) 121 (57.6)	21(23.6) 34 (28.1)	68 (76.4) 87 (71.9)
Surgical procedure			
ENT Ophthalmic Abdominal Orthopedic Neurosurgery	25 (11.9) 34 (16.2) 75 (35.7) 34 (16.2) 41 (20)	12 (48) 4 (11.8) 17 (22.7) 6 (17.6) 16 (38.1)	13 (52) 30 (88.2) 58 (77.3) 28 (82.4) 26 (61.9)
Airway device			
Endotracheal tube Laryngeal mask airway Facemask	136 (64.8) 46 (21.9) 28 (13.3)	40 (29.4) 8 (17.4) 7 (25.0)	96 (70.6) 38 (82.6) 21 (75.0)
Induction of anesthesia			
Intravenous Inhalation	199 (94.8) 11 (5.2)	51 (22.6) 4 (36.4)	148 (74.4) 7 (63.6)
Muscle relaxant			
Yes No	120 (57.1) 90 (42.9)	32 (26.7) 23 (25.6)	88 (73.3) 67 (74.4)
Time of extubation			
Awake extubation Deep extubation	175 (96.2) 7 (3.8)	46 (26.3) 2 (28.6)	129 (73.7) 5 (71.4)
Intraoperative blood loss			
<100 ml 100–500 ml ≥ 500 ml	72 (34.7) 99 (46.2) 41 (19.5)	18 (25) 29 (29.9) 8 (19.5)	54 (75) 29 (29.9) 33 (80.5)

*IQR, Inter-quartile Range*.

**Figure 1:**
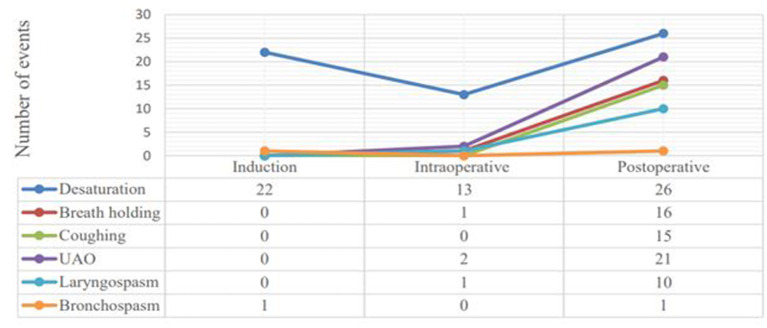
Line graphs showing the frequency of respiratory adverse events among distinct phases of the perioperative period in pediatric surgical patients at UoGCSH and TGSH, Northwest Ethiopia; March 1–May 30, 2020 (*N* = 20).

Out of 210 pediatric patients, 55 (26.2%, CI: 20.5–30.9%) had developed PRAEs. We have observed 129 episodes of PRAEs and desaturation was the commonest adverse event which occurred 61 (47.3%) times; followed by partial upper airway obstruction 21 (16.3%), breath holding 17 (13.2%), persistent coughing 15 (11.6%), laryngospasm 11 (8.5%) and bronchospasm 2 (1.6%). Of 129 episodes of PRAEs, 89 (69.0%) occurred in the postoperative period while 23 (17.8) were occurred during induction of anesthesia, and 17 (13.2%) during the maintenance phase (Figure 1).

Among four pediatric surgical patients who had family history of asthma, bronchospasm occurred in the two of them. One was during induction of anesthesia and the other was after extubation. Fisher-exact test has showed the significant association between family history of asthma and per ioperative bronchospasm. However, none of the children had self-history of asthma. The relations of independent variables with outcome variables in Pearson's Chi-squared and Fisher-exact tests are presented (Table 3). Regarding severity of PRAEs, 10 (4.8%) patients had serious desaturation (SpO_2_ <80%) and 1 (0.5%) patient had continuous coughing, 3 (1.4%) patients had complete airway obstruction which required muscle relaxant, and 9 (4.3%) patients had laryngospasm which was effectively managed by the applications of simple airway maneuvers and positive airway pressure (Table 4).

**Table 3:** Pearson Chi-squared and Fisher-exact tests; factors associated with perioperative respiratory adverse events among pediatric surgical patients underwent surgery under general anesthesia at UoGCSH and TGSH, Northwest Ethiopia; March 1–May 30, 2020 (*N* = 210).

**Variables**	**Frequency *n* (%)**	**Desaturation *n* (%)**	**Breath holding n (%)**	**Coughing *n* (%)**	**Airway obstruction *n* (%)**	**Laryngospasm *n* (%)**	**Bronchospasm *n* (%)**
**Age**							
≤ 1year > 1year	52 (24.8) 158 (75.2)	22 (44.0)^a^ 28 (56.0)	8(50.0) 8 (50.0)	8 (53.3)^b^ 7 (46.7)	5 (23.8) 16 (76.2)	5 (41.7) 7 (58.3)	1 (1.9) 1 (0.6)
**ASA**							
≥ 3 ≤ 2	21 (10) 189 (90)	14 (28.0)^a^ 36 (72.0)	2 (12.5) 14 (87.5)	5 (33.3) 10 (66.7)	8 (38.1) 13 (61.9)	2 (16.7) 10 (83.3)	0 2 (1.1)
**URTI**							
Yes No	23 (11) 187 (89)	17 (34.0)^a^ 33 (66.0)	4 (25.0) 12 (75.0)	6 (40)^b^ 9 (60)	6 (28.5)^b^ 15 (71.5)	5 (41.7)^b^ 7 (58.3)	0 2 (1.1)
**Attempts**							
≥3 ≤ 2	9 (4.30) 201 (95.7)	7 (14.0)^b^ 38 (86.0)	2 (12.5) 14 (87.5)	1 (6.7) 14 (93.3)	0 21 (100)	3 (25)^b^ 9 (75)	1 (50) 1 (50)
**Secretion**							
Yes No	26 (12.4) 184 (87.6)	15 (30.0)^a^ 35 (70.0)	5 (31.2)^b^ 11 (68.8)	8 (53.3)^b^ 7 (46.7)	9 (42.8)^b^ 12 (57.2)	5 (41.7)^b^ 7 (58.3)	0 2 (1.1)
**Procedures**							
Airway Non-airway	25 (11.9) 185 (88.1)	10 (20)^a^ 40 (80)	3 (18.75) 13 (81.2)	4 (26.6) 11 (73.4)	7 (33.3) 14 (66.7)	4 (33.3) 8 (66.7)	0 2 (1.1)
**Family history of Asthma**							
Yes No	4 (1.9) 206 (98.1)	4 (8.0) 46 (92.0)	0 16 (100)	0 15 (100)	1 (4.7) 20 (95.3)	0 12 (100)	2 (50.0)^b^ 0

a *Significant in Pearson Chi-square test*.

b *Significant in Fisher-exact test.*

**Table 4:** Severity of perioperative respiratory adverse events among pediatric surgical patients underwent surgery under general anesthesia at UoGCSH and TGSH, Northwest Ethiopia; March 1–May 30, 2020 (*N* = 210).

**Variables**	**Severity Score** ***n*** (%)			
	**1**	**2**	**3**	**4**
SpO_2_	160 (76.2)	22 (10.5)	18 (8.6)	10 (4.8)
Coughing (*n*)	194 (92.4)	2 (1)	14 (6.7)	0
Breath holding (seconds)	195 (92.9)	9 (4.3)	5 (2.4)	1 (0.5)
Laryngospasm	177 (84.2)	21 (10)	9 (4.3)	3 (1.4)
Bronchospasm	208 (99)	1 (0.5)	1 (0.5)	0
Secretions	184 (87.6)	0	15 (7.1)	11 (5.2)

In bivariate binary logistic regression analysis, comorbidity, emergency surgery, multiple attempts of tracheal intubation (≥ 3 attempts), light anesthesia, perioperative opioid use and duration of surgery ≥ 60 min were found associated with PRAEs. The final multivariate binary logistic regression analysis demonstrated that age <1 year, URTIs (recent or active), secretions in the upper airways, ASA ≥ 3 and airway related surgery were associated with PRAEs.

Among pediatric surgical patients, those who had URTIs were more than seven times likely to develop PRAEs (AOR: 7.6, CI: 1.9–30.2, p: 0.004). The likelihood of PRAEs to occur in infants was 3.6 times than older pediatric surgical patients (AOR: 3.6, CI: 1.3–10.0, p: 0.012). Interestingly, repeated attempts of tracheal intubation were noticed among infants (66.6 vs. 33.3%, p: 0.008). ASA physical status of three and above increases the occurrence of PRAEs by more than 5 folds (AOR: 5.2, CI: 1.9–22.9, *p*: 0.029). Pediatric surgical patients who underwent airway related procedures had developed PRAEs more frequently compared to their counterparts (AOR: 6.0, CI: 1.5–24.1, *p*: 0.012). Having moderate to copious oropharyngeal secretions was found associated with the occurrence of PRAEs (AOR: 4.8, CI: 1.4–15.9, *p*: 0.011). The results of this study claimed that prematurity, premedication, induction agents, types of airway devices and experiences of the anesthetists were not associated with PRAEs (Table 5).

**Table 5:** Bivariate and multivariate binary logistic regression analysis: factors associated with PRAEs in pediatric surgical patients underwent surgery under general anesthesia in UoGCSH and TGSH, Northwest Ethiopia; March 1–May 30, 2020 (*N* = 210).

**Variables**	**PRAEs**		**Odds ratio (95% CI)**		***p*-values**
	**Yes *n* (%)**	**No *n* (%)**	**Crude**	**Adjusted**	
**Age**					
<1 year ≥ 1 year	22 (42.3) 33 (20.9)	30 (57.7) 125 (79.1)	2.8 (1.4, 5.4) 1	3.6 (1.3, 10.0) 1	0.012
**ASA class**					
≥ 3 ≤ 2	15 (71.4) 40 (21.2)	6 (28.6) 149 (78.8)	9.31 (3.4, 25.8) 1	5.2 (1.2, 22.9) 1	0.029
**Urgency of the procedure**					
Emergency Elective	39 (34.2) 16 (16.7)	75 (65.8) 80 (83.3)	2.6 (1.3, 5.0) 1	1.41 (0.5, 3.7) 1	0.48
**URTIs**					
Yes No	17 (73.9) 38 (20.3)	6 (26.1) 149 (79.7)	11.1 (4.1, 30.1) 1	7.5 (1.9, 30.2) 1	0.004
**Surgical procedures**					
Airway related Non-airway related	12 (48.0) 43 (23.2)	13 (52.0) 142 (76.8)	3.0 (1.3, 7.2) 1	6.0 (1.5, 24.1) 1	0.012
**Secretions in the upper airway**					
Moderate to copious None to minimal	18 (69.2) 37 (20.1)	8 (30.8) 147 (79.9)	8.9 (3.6, 22.2) 1	4.78 (1.4, 18.8) 1	0.011
**Light anesthesia**					
Yes No	10 (41.7) 45 (24.2)	14 (58.3) 141 (75.8)	2.2 (0.9, 5.4) 1	2.6 (0.7, 9.8) 1	0.14
**Intubation attempts**					
≥ 3 ≤ 2	7 (77.8) 42 (24.1)	2 (22.2) 132 (75.9)	11 (2.2, 55.0) 1	3.9 (0.5, 28.0) 1	0.17
**Duration of surgery (hour)**					
≥ 1 <1	39 (37.7) 16 (18.4)	84 (68.3) 71 (81.6)	2 (1.1, 4.0) 1	2.0 (0.7, 5.9) 1	0.19

*NB: p-values are from multivariate binary logistic regression analysis*.

## Discussion

The overall prevalence of PRAEs among pediatric surgical patients who underwent surgery under general anesthesia at UoGCSH and TGCSH was high (26.2%). This result was congruent with a study done by Mamie et al. in which it was 21% (1). A systematic review also has revealed that the prevalence of PRAEs was in the ranges of 8–21% (2). Similarly, Ramgolam et al. concluded that the prevalence of PRAEs was 26–43% (7). The differences can be explained by the inclusion of only patients who had higher risk of developing PRAEs in the former study.

Prevalence of PRAEs was higher in our study compared to previous study in which it was 15% (15). The deviations could be due to inclusion of infants in our study as we noticed an increased occurrence of PRAEs in infants compared to older children. In addition, previous multiple studies verified lower prevalence of PRAEs compared to the current study (2.8, 5.7, and 17.8%) (27–29). Higher prevalence in our study might be due to the inclusion of patients with higher ASA physical status (≥ 3), recovery of pediatrics patients at post-anesthesia care unit that were not staffed with professionals with special pediatric trainings and ill-equipped recovery area. A retrospective multicenter study done among 25,098 patients showed that the prevalence of PRAEs was 4.6%. The discrepancies might be explained by the incorporation of a large numbers of emergency patients in our study (54.3 vs. 27.4%) (12). In-addition, differences in the definitions of PRAEs, study design and sample sizes might explain it.

Oxygen desaturation was occurred in 50 (23.8%) patients which was the commonest PRAE, particularly among patients who had URTIs and airway secretions. This finding was similar with a multicenter study in terms of the overall occurrence. However, it was more frequent intraoperatively compared to emergence (12). In our study, desaturation was common after tracheal extubation. Anesthetic agents are known for diminishing respiratory drive that can results in hypoxemia in the postoperative period (30). Furthermore, almost all types of PRAEs can lead to desaturation.

Persistent coughing and upper airway obstruction were occurred in 15 (7.1%) and 21 (10%) patients respectively. In a previous study, the prevalence of upper airway obstruction was 3.6–9% (2). Budic and Simic have reported that persistent coughing was occurred only in three out of 682 patients (0.004%). The differences in definitions of persistent coughing might cause the inconsistencies. In the current study, persistent coughing was defined as coughing that sustained for 5 s; however, others have defined it as coughing that sustained for 5–10 s (28, 29).

Breathe holding was occurred in 16 (7.6%) patients and it was higher compared to a study done by Tait et al. (23). A large numbers of infant patients [52 (24.8%)] were included in our study. Younger children have a tidal volume that occurs at the same volume as a closing volume; therefore, the terminal bronchioles close easily causing apnea and desaturation (30). Furthermore, neuromuscular blocking agents were used extensively in 120 (57.1%) patients. This might contribute for higher incidence of breath holding among our patients (31). In our study areas, there was no any qualitative monitoring of residual neuromuscular blockade.

The prevalence of laryngospasm was 12 (5.7%) and consistent with a systematic review which claimed 0.1–16%. Particularly, it was 4% in general pediatric population (2). The commonest time of occurrence was extubation. Similarly to previous studies, laryngospasm was associated with presence of airway secretions and URTIs (22, 32). Family history of asthma was associated with perioperative bronchospasm. The phenomenon can be explained by bronchial hyper-responsiveness in children born from parents who have asthma (33).

Children that presented with recent or active sign and symptoms of URTIs were found to have increased risk of developing PRAEs (73.9%). The higher risk of PRAEs during URTIs can be justified by the morphologic damage to the epithelium and mucosa of the respiratory tract after infections and make the airway sensitive to potentially irritant anesthetic gases and secretions that result in activation of irritant receptors and contractions of airway smooth muscles (30). A study has showed that there was increased incidence of PRAEs in patients with an active URTIs than those who had recent URTIs (2–4 weeks before the procedure) (34). The effects of URTIs on the airway could last for several weeks (3). In our study there was no significant difference in the occurrence of PRAEs whether the URTI is recent or active (84.6 vs. 60%, *p*: 0.34). The occurrence of PRAEs was significantly higher immediately after tracheal extubation than induction and maintenance phases (30).

Infants were more vulnerable to develop PRAEs than older patients (8, 20). Desaturation occurs more frequently in infants (5, 8). Infants have higher oxygen demand and low oxygen reserves which make them risky to PRAEs such as hypoxia. Breath holding was common in this group of patients due to immature respiratory center. Additionally, we have noticed that multiple attempts of tracheal intubation in infants than older patients (66.7 vs. 33.3%, *p*: 0.008). A previous study concluded that multiple intubation attempts were associated with PRAEs (20).

There was higher occurrence of PRAEs among patients with higher ASA physical status (≥ 3) and this is supported by previous studies (4, 11). In developing countries, anesthetists frequently encounter patients with poorly treated comorbidities which could lead to the occurrence of PRAEs (4, 5, 35). The majority of the patients were underwent emergency surgery 114 (54%); therefore, poor preoperative optimization might contribute for higher incidence of PRAEs (35).

The risk of developing PRAEs was increased by 6-folds when a surgical procedure involves the airway. Airway related procedures were associated with frequent desaturation and upper airway obstruction. Our finding was supported by previous multicenter prospective studies (3, 5, 36). Children who need airway related surgery commonly have chronic airway inflammation and bronchial hypersensitivity that could result in PRAEs. Surgical instrumentation and manipulation of the airway can lead to laryngeal reflex response (2). Moderate to copious airway secretions was found associated with PRAEs, particularly with desaturation and partial upper airway obstruction. Soiling of the airway which necessitate repeated suctioning can potentiate airway hyper-reactivity (23).

A multicenter study done by Habre et al. including 261 hospitals across Europe has concluded that the experience of the team of anesthesia providers was a very important factor that determines the safety and the rate of adverse events in the pediatric anesthesia practices (37). Our study have not showed this association. The discrepancy might be due to smaller sample size and setup differences as only two hospitals included in the current study done in a developing country.

The study was the first for its type in our country and we believe that it can be a foundation for future studies in the field; especially in resource-limited settings. Hypoventilation and residual neuromuscular blockade were not measured due to the lack of the devices in the post-anesthesia care units of the hospitals. Smaller sample size due to COVID-19 pandemic is another limitation of this study.

## Conclusions

There was high prevalence of perioperative respiratory adverse events among pediatric surgical patients. The postoperative period was the most critical time for the occurrence of PRAEs and desaturation was the commonest adverse event. Age <1 year, URTIs (recent or active), secretions in the upper airways, ASA ≥ 3 and airway surgery were significantly associated with adverse events. Clinicians should perform effective risk assessment, optimization and preparation for the management of perioperative respiratory adverse events.

## Data Availability Statement

The raw data supporting the conclusions of this article will be made available by the authors, without undue reservation.

## Ethics Statement

The studies involving human participants were reviewed and approved by University of Gondar. Written informed consent to participate in this study was provided by the participants' legal guardian/next of kin.

## Author Contributions

DW and YWB have conceptualized the study, objectives, and manuscript preparation. DW has developed the proposal. YWB, WC, HA, and MW have criticized the proposal. All authors had participated in the data and statistical analyses and read and approved the final manuscript.

## Funding

This work was supported by University of Gondar and Health Bureau of Amhara National Regional State, Ethiopia.

## Conflict of Interest

The authors declare that the research was conducted in the absence of any commercial or financial relationships that could be construed as a potential conflict of interest.

## Publisher's Note

All claims expressed in this article are solely those of the authors and do not necessarily represent those of their affiliated organizations, or those of the publisher, the editors and the reviewers. Any product that may be evaluated in this article, or claim that may be made by its manufacturer, is not guaranteed or endorsed by the publisher.

## Abbreviations

AOR: Adjusted Odds Ratio
ASA: American Society of Anesthesiologist
COVID-19: Corona Virus Disease-2019
GA: General Anesthesia
LMA: Laryngeal Mask Airways
PRAEs: Perioperative Respiratory Adverse Events
SPO2: Peripheral Oxygen Saturation
SPSS: Statistical Package for Social Science
TGSH: Tibebe-Ghion Specialized Hospital
UoGCSH: University of Gondar Comprehensive and Specialized Hospital
URTIs: Upper Respiratory Tract Infections.
